# Leser–Trélat Sign as an Initial Manifestation of Synchronous Prostate and Bladder Cancer

**DOI:** 10.7759/cureus.93325

**Published:** 2025-09-27

**Authors:** Alper Okur, Kadir Can Şahin, Zehra Sahin, Mehmet Hamza Gultekin, Bulent Onal

**Affiliations:** 1 Urology, Cerrahpasa Faculty of Medicine, Istanbul University - Cerrahpasa, Istanbul, TUR; 2 Urology, Istanbul Bayrampasa State Hospital, Istanbul, TUR; 3 Medical Pathology, Istanbul Gaziosmanpasa Training and Research Hospital, Istanbul, TUR

**Keywords:** leser-trélat sign, paraneoplastic skin disease, prostate adenocarcinoma, seborrheic keratoses, urothelial bladder cancer

## Abstract

The Leser-Trélat sign, marked by the sudden onset and rapid proliferation of seborrheic keratoses, is a rare paraneoplastic phenomenon associated with internal malignancies. This report presents a case of a 75-year-old male with a history of hypertension and diabetes who presented with an elevated PSA level and was subsequently diagnosed with synchronous prostate adenocarcinoma and high-grade invasive urothelial carcinoma. Prior to the oncologic diagnoses, the patient exhibited cutaneous lesions consistent with the Leser-Trélat sign, which raised clinical suspicion for an underlying malignancy. The patient's treatment course comprised transurethral resection of the bladder tumor, complete intravesical Bacillus Calmette-Guérin therapy, radical prostatectomy, and subsequent combined androgen deprivation therapy with salvage radiotherapy due to persistent postoperative PSA elevation. The stabilization of the patient's dermatologic manifestations following oncologic treatment supports the hypothesis of a paraneoplastic origin. The Leser-Trélat sign, while infrequent and debated, may serve as an early indicator of malignancy, particularly when observed with atypical seborrheic keratosis distribution. Although the pathogenesis remains incompletely understood, molecular associations such as FGFR3 mutations have been implicated. The co-occurrence of this sign with synchronous genitourinary malignancies underscores the importance of a multidisciplinary approach to diagnosis and management. Further investigation is warranted to elucidate the precise underlying mechanisms of this rare phenomenon and its potential role in oncologic detection.

## Introduction

Paraneoplastic syndromes are defined as hormonal, neurological, or hematological disturbances and as clinical and biochemical imbalances related to the presence of malignancies, with no direct association with primary tumor invasion or metastasis [1]. Skin manifestations can sometimes be the first sign of an occult malignancy [2]. Among the various cutaneous paraneoplastic manifestations, the sign of Leser-Trélat is a rare and controversial entity characterized by the abrupt eruption and rapid increase in the number and size of seborrheic keratoses with an internal malignancy [3]. Its true incidence remains uncertain, as only several hundred cases have been reported in the literature, and controversy persists as to whether it represents a distinct paraneoplastic entity or a coincidental finding in elderly patients with cancer. Nevertheless, recognition of this sign is clinically relevant, as it may serve as an early dermatologic clue to an otherwise occult malignancy.

Although the association between Leser-Trélat sign and internal malignancies has been described, its etiopathogenesis remains poorly understood, and its clinical significance is debated. Previous reports have documented the association of Leser-Trélat sign with various malignancies, including, in isolated instances, prostate adenocarcinoma and transitional cell carcinoma of the bladder [4-7].

This case report aims to describe a unique presentation involving Leser-Trélat sign, occurring in the context of synchronous primary malignancies of the bladder and prostate. This presentation is particularly noteworthy due to the rare coexistence of bladder and prostate tumors, with the Leser-Trélat sign being an exceedingly rare occurrence. The report emphasizes the clinical implications of recognizing Leser-Trélat sign in such complex scenarios, the importance of thorough clinical examination, the potential role of specific molecular markers, and the need for further investigation to elucidate the complex relationship between this paraneoplastic dermatosis and underlying malignancy.

## Case presentation

A 75-year-old male patient with a medical history of hypertension and type 2 diabetes mellitus was referred to our clinic following the detection of an elevated prostate-specific antigen (PSA) level of 11.3 ng/mL by his primary care physician. Eight years prior, he had undergone transurethral resection of the prostate (TUR-P) at another center for benign prostatic hyperplasia. Initial clinical evaluation included a digital rectal examination, which revealed a solid nodule in the left lobe, suspicious for malignancy. Consequently, multiparametric magnetic resonance imaging (mpMRI) of the prostate was ordered.

**Figure 1:**
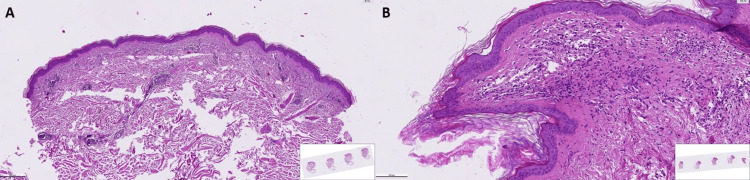
Histopathological features of the biopsy specimen (H&E staining). (A) Perivascular inflammation composed of lymphocytes, histiocytes, and a few eosinophils (x6.1). (B) Extravasation of erythrocytes in the perivascular region (x19.7).

**Figure 2:**
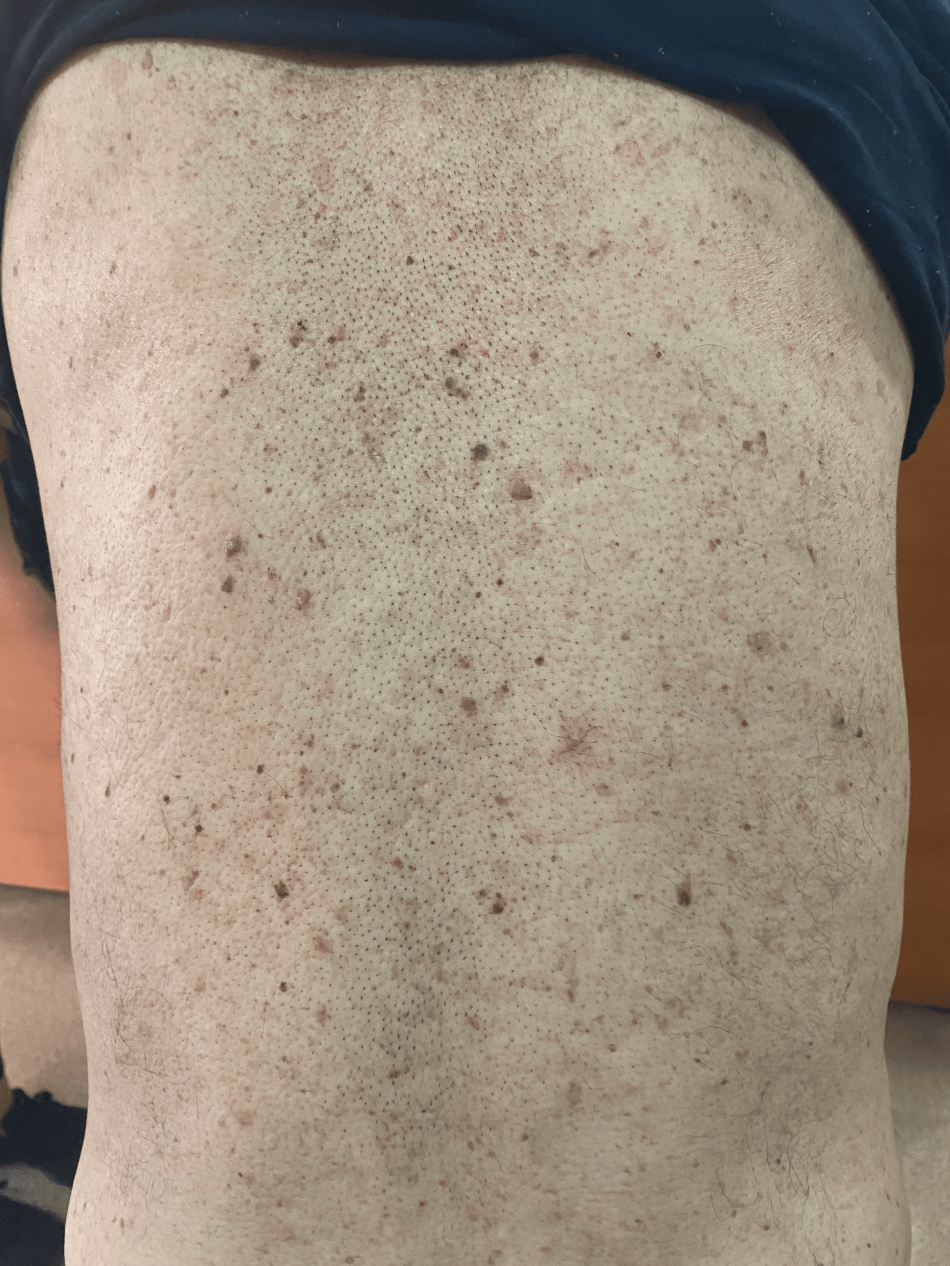
Rapidly increasing number and size of seborrheic keratoses detected in the patient, developing one year after the biopsy in which pigmented purpuric dermatosis had been diagnosed.

Further inquiry into the patient's medical history revealed that a biopsy of newly developed cutaneous lesions on the back, groin, and thighs had been performed one year prior to the sudden onset of seborrheic keratoses. Histopathological analysis of these lesions demonstrated mild hyperkeratosis and focal spongiosis in the epidermis, with a predominantly perivascular infiltration extending into the interstitium, composed of lymphocytes, histiocytes, and rare eosinophils. Slight fibroblastic activity and mild vascular proliferation were also noted in the upper dermis. These findings were suggestive of a pigmented purpuric dermatosis (Figure 1). The patient reported a recent exacerbation of these lesions, prompting consideration of the Leser-Trélat sign, which was subsequently confirmed following a dermatological consultation (Figure 2).

**Figure 3:**
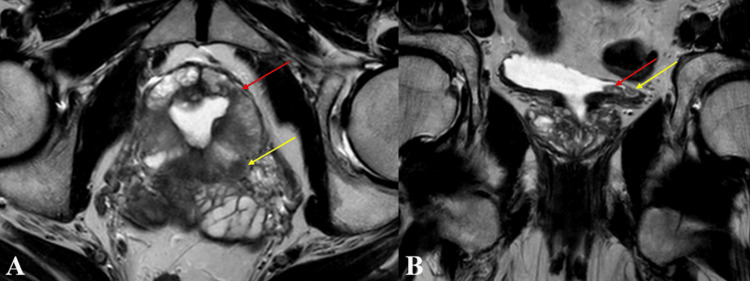
A: PI-RADS 4 lesions and distorted and dilated prostatic urethra secondary to prior transurethral resection of the prostate detected on multiparametric prostate MRI (red arrow: 7.5 mm lesion in the posterolateral left peripheral zone at the midgland level, yellow arrow: 3.5 mm lesion in the anterior peripheral zone). B: Bladder tumor detected on multiparametric prostate MRI (red arrow: 10x5 mm lesion, yellow arrow: 6x3.5 mm lesion).

The patient's mpMRI, performed one week later, revealed two PI-RADS 4 lesions with T2-hypointensity, apparent diffusion coefficient (ADC)-hypointensity, diffusion restriction, and early contrast enhancement, located in the posterolateral left peripheral zone at the midgland level (7.5 mm) and the anterior peripheral zone (3.5 mm) (Figure 3). The prostatic urethra exhibited dilation and distortion due to prior TUR-P. Additionally, two T2-hypointense, diffusion-restricting, lumen-protruding lesions were observed in the bladder, measuring 10x5 mm at the base and 6x3.5 mm at the left lateral wall (Figure 3).

In December 2021, the patient underwent a complete transurethral resection of bladder tumor (TUR-BT), and the pathological evaluation confirmed pT1 high-grade invasive urothelial carcinoma. Subsequently, in January 2022, a transrectal MRI-fusion biopsy of the prostate revealed Gleason 4+3 acinar adenocarcinoma in five of 16 quadrants. A Ga-68 prostate-specific membrane antigen positron emission tomography (PSMA-PET) scan demonstrated focal increased uptake in the prostate gland, with no evidence of additional involvement. Given the synchronous tumors, the patient's case was reviewed in a multidisciplinary uro-oncology meeting to determine the optimal management strategy. Following shared decision-making with the patient, a treatment plan was formulated, prioritizing the bladder cancer with the initiation of Bacillus Calmette-Guérin (BCG) therapy. Cystoprostatectomy was considered in the event of intravesical BCG treatment failure, while radical prostatectomy was planned if the bladder tumor responded to intravesical BCG.

Four weeks following the initial TUR-BT, a repeat TUR-BT demonstrated no residual tumor. The patient then completed six doses of BCG induction therapy. Following BCG induction, cystoscopic surveillance revealed no evidence of tumor recurrence, and the patient tolerated BCG maintenance therapy without complications. In January 2023, the patient underwent robot-assisted laparoscopic radical prostatectomy with bilateral extended pelvic lymph node dissection. Pathological evaluation of the surgical specimen revealed pT2cN0M0 - R0, Gleason 4+3=7 acinar adenocarcinoma.

Postoperatively, persistent elevation of PSA levels was observed at the first and third-month follow-up (1.87 and 1.82 ng/mL, respectively). Consequently, the patient was re-evaluated in the multidisciplinary uro-oncology meeting, and salvage radiotherapy was recommended. Androgen deprivation therapy was initiated, and curative salvage radiotherapy, consisting of 66 Gy in 33 fractions to the prostatic bed, was planned for August 2023.

Currently, the patient, having completed maintenance intravesical BCG therapy, is under active surveillance for both prostate and bladder cancer, with no evidence of recurrence. The cutaneous lesions identified at the time of diagnosis and those that subsequently developed have remained stable without further progression following oncological treatment and remain stable.

## Discussion

The concurrence of synchronous bladder and prostate tumors represents a complex clinical entity, further compounded by the manifestation of the Leser-Trélat sign. It is important to highlight that, while previous reports have documented the association of the Leser-Trélat sign with either prostate cancer or bladder [4-7], our case presents a unique scenario involving synchronous tumors of both the bladder and prostate, further underscoring the complexity of this paraneoplastic presentation.

Paraneoplastic skin manifestations can be associated with underlying urologic malignancies [1]. Some examples include Bazex syndrome, Sweet syndrome, dermatomyositis, acquired ichthyosis, and erythema gyratum repens. The sign of Leser-Trélat is defined by the sudden appearance and rapid increase in the number and size of seborrheic keratoses, which are benign skin tumors that typically appear as waxy, raised lesions with a slightly granular surface, with a distinctive "stuck-on" appearance [3,8]. While seborrheic keratoses are common, benign, and slow-growing in the elderly, the Leser-Trélat sign is distinguished by their abrupt onset or a rapid increase in size and number, often in association with pruritus and other dermatological conditions, such as acanthosis nigricans. Although the pathogenesis of Leser-Trélat sign remains controversial, and some authors consider the association of seborrheic keratoses with internal malignancies as coincidental, the recognition of this sign should prompt a thorough search for an underlying malignancy, especially associated with lymphoproliferative disorders, gastrointestinal or pulmonary carcinomas [8-10].

The conventional topography of seborrheic keratoses typically encompasses the thoracic and dorsal regions, followed by the extremities, face, abdomen, cervical region, and axillae [3,8]. In the presented case, the observed involvement of the buttocks, thighs, and groin deviates from the typical distribution, thereby raising clinical suspicion for a potential paraneoplastic phenomenon. The recognition of such atypical localizations is of paramount importance in the context of comprehensive dermatologic assessment. Clinical observations have demonstrated a discernible correlation between the progression of seborrheic keratoses and the status of the underlying malignancy. The cessation or regression of lesions following definitive oncologic intervention, as reported in previous studies, lends support to the paraneoplastic nature of this dermatosis.

Fibroblast growth factor receptor 3 (FGFR3) mutations have been implicated in the pathogenesis of both bladder cancer and seborrheic keratoses [8-14]. Another study reported that the FGRF3 gene does not play a central role in the pathogenesis of prostate cancer, but is significantly associated with a subgroup of low-grade prostate tumors and the presence of other tumors, particularly those arising in the bladder and skin [15]. While not investigated in this specific case, the potential contribution of FGFR3 signaling pathways to the paraneoplastic induction of Leser-Trélat sign warrants further investigation in future studies. This case underscores the indispensable role of meticulous physical examination in patients presenting with the Leser-Trélat sign. Comprehensive clinical evaluation, coupled with targeted complementary investigations, is crucial for the early detection of underlying malignancies. The recognition of paraneoplastic dermatoses may facilitate timely cancer diagnosis and potentially improve patient outcomes.

The association between Leser-Trélat sign and genitourinary malignancies, although documented, remains a relatively infrequent clinical occurrence [4-7]. Optimal management of patients with synchronous bladder and prostate tumors presenting with Leser-Trélat sign mandates a multidisciplinary approach, involving urologic oncology, dermatopathology, and supportive care. The differential diagnosis of Leser-Trélat sign necessitates the consideration of other dermatologic entities characterized by multiple seborrheic keratoses, as well as other paraneoplastic skin conditions. In contrast to seborrheic keratosis in a patient without underlying malignancy, histopathological analysis of a biopsied seborrheic keratosis from a patient suspected of exhibiting the sign of Leser-Trélat does not reveal any discernible differences [10].

Although a coincidental association between seborrheic keratoses and internal malignancies cannot be entirely excluded, the abrupt onset or accelerated growth of seborrheic keratoses should prompt a high index of suspicion for underlying malignancy. This case contributes to the existing scholarly literature by providing a detailed account of a rare and complex clinical presentation of Leser-Trélat sign associated with synchronous bladder and prostate tumors. Further investigations are warranted to elucidate the precise molecular mechanisms underlying this paraneoplastic phenomenon and to establish an evidence-based clinical management algorithm.

## Conclusions

This case report describes a rare and complex presentation of Leser-Trélat sign in association with synchronous primary malignancies of the bladder and prostate. The sign of Leser-Trélat, although controversial, has been associated with various internal malignancies, with previous reports documenting isolated occurrences in the context of either prostate or bladder cancer. The present case distinguishes itself by the synchronicity of the bladder and prostate tumors, highlighting the potential of the Leser-Trélat sign to manifest in intricate oncologic scenarios.

The recognition of paraneoplastic dermatoses, such as the Leser-Trélat sign, is crucial, as it may prompt investigations, leading to the early detection and treatment of underlying malignancies, potentially improving patient prognosis. Clinicians should be cognizant of atypical clinical presentations and maintain a high index of suspicion for paraneoplastic phenomena. Further research is warranted to elucidate the precise mechanisms underlying the Leser-Trélat sign, particularly in the context of synchronous malignancies, and to establish definitive diagnostic and management strategies.
